# Emerging role of m6A modification in ovarian cancer: progression, drug resistance, and therapeutic prospects

**DOI:** 10.3389/fonc.2024.1366223

**Published:** 2024-03-13

**Authors:** Shahil Alam, Pankaj Kumar Giri

**Affiliations:** Faculty of Life Sciences and Biotechnology, South Asian University, New Delhi, India

**Keywords:** m6A RNA modifications, epitranscriptomics, cancer therapy, ovarian cancer, drug-resistance

## Abstract

Ovarian Cancer (OC) ranks as a prominent contributor to mortality among female reproductive system associated cancers, particularly the prevalent subtype epithelial Ovarian Cancer (EOC). Despite advancements in treatment modalities, the prognosis for OC patients remains grim due to limitation of current therapeutic methodology such as high cytotoxicity of chemotherapeutic agents and tumor relapse making existing chemotherapy ineffective. Recognizing the limitations of a broad-spectrum approach to treating OC, a shift toward targeted therapies aligning with unique molecular features is imperative. This shift stems from an incomplete understanding of OC’s origin, distinguishing it from extensively researched malignancies such as cervical or colon cancer. At the molecular level, postsynthetic modifications—DNA, RNA, and protein—shape transcriptional, posttranscriptional, and posttranslational processes. Posttranscriptional regulatory mechanisms, including RNA modifications are termed epitranscriptomic and play critical roles in this process. For more than five decades, 100+ RNA post-synthetic modifications, notably N6-methyladenosine (m6A), most prevalent RNA modification in mammals, dynamically regulate messenger RNA (mRNA), and non-coding RNA (ncRNA) life orchestrated via writers, erasers, and readers. The disruption of m6A modifications are found in several cancers, including OC, underscores pivotal role of m6A. This review focused on m6A modifications in coding and non-coding RNAs, emphasizing their role as prognostic markers in OC and their impact on development, migration, invasion, and drug resistance. Additionally, RNA-modified regulators have been explored as potential molecular and therapeutic targets, offering an innovative approach to combatting this challenging malignancy.

## Graphical Abstract

Created with BioRender.com, and Corel Draw

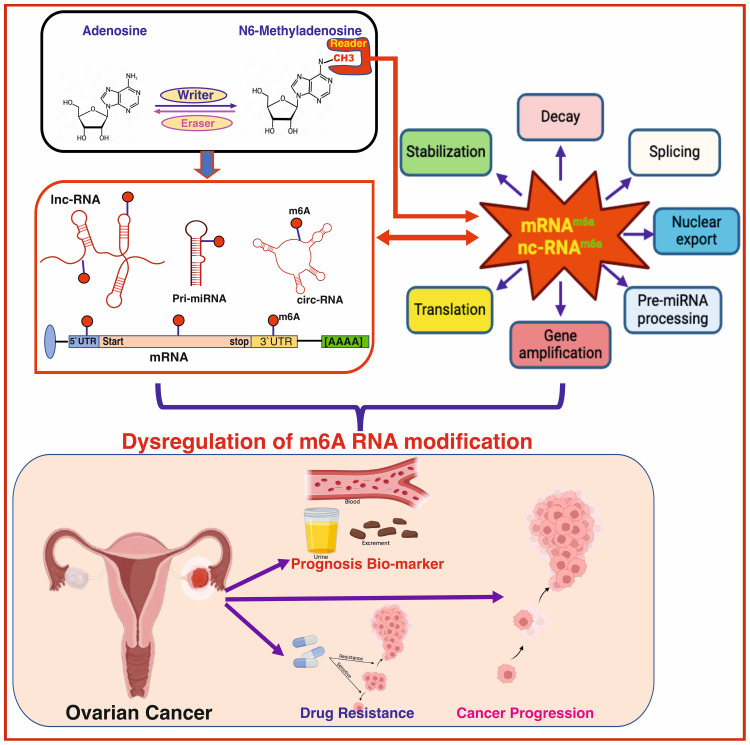

## 1 Introduction

Ovarian Cancer (OC) stands out as a prominent gynecologic malignancy, holding the first position of the fatality-inducing factor among tumors affecting the female reproductive system (1).

OC ranks as 18^th^, and 14^th^ in term of incidence, and mortality respectively among different cancers (2). The composition of ovarian tumor tissue is highly intricate, with the ovary having the highest diversity of primary tumor types among all organs in the body. Various ovarian cancers exhibit significant differences in histological structure and biological behavior. The primary histological categories of OC include epithelial OC (EOC), sex cord stromal OC, and germ cell OC. Among these, EOC are the most prevalent, constituting approximately 50%-70% of cases. EOC, based on tumor cell histology, is further categorized into serous (52%), endometrioid (10%), mucinous (6%), clear cell (6%), and other diverse types (3, 4).

**Figure 1:**
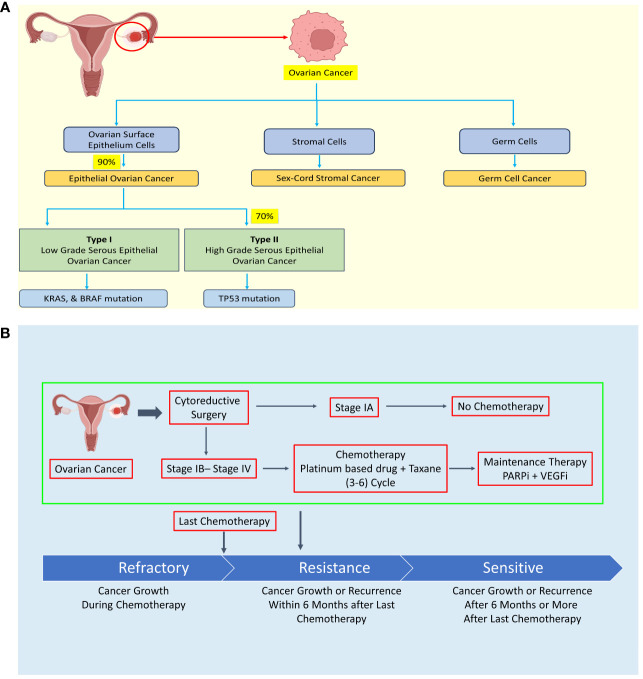
Diagram illustrating the classification of ovarian cancer (OC) according to histology, treatment approaches, and the development of resistance mechanisms. **(A)** Ovarian cancer is categorized into epithelial OC, sex-cord stromal cancer, and germ cell cancer based on histological characteristics. **(B)** Treatment of ovarian cancer typically involves debulking surgery followed by chemotherapy, with the exception of stage IA cases. However, some OC patients may develop chemoresistance during chemotherapy or experience recurrence within six months or more after their last chemotherapy session. Created with BioRender.com.

Based on clinicopathological and molecular genetic features, EOC is further classified into type I and type II, each exhibiting distinct characteristics. Type I tumors typically exhibit slow growth, are predominantly diagnosed at stage I clinically, and have a favorable prognosis. In contrast, type II tumors grow rapidly, are often diagnosed at advanced stages, and carry a poorer prognosis (see **Figure 1A**) (5–8).

OC often asymptomatic early; symptoms emerge late, usually in advanced stages with widespread metastasis to uterus, bilateral adnexa, and pelvic organs (9–11). Despite efforts to screen using serum cancer antigen 125 (CA-125) and transvaginal ultrasound (TVUS), there is no significant reduction in ovarian cancer mortality (12). Presently, no single screening test is universally endorsed for OC. The intricates molecular mechanism contributing to tumor growth in OC and potential therapeutic targets remain largely unknown (13, 14).

The high cytotoxicity and resistance of chemotherapy drug are major hurdle in OC therapeutic strategy (see **Figure 1B**). The four major chemotherapy drugs viz. platinum-based drug, paclitaxel, PARP inhibitors, and VEGF inhibitors exhibit drug resistance sooner or later leading to failure of chemotherapy and seeking of alternative strategy such as combinatorial chemotherapy revealing urgent need of specific, low toxic drug to treat OC patient including relapse patient (15–22). The treatment is not specific to OC leading to high cytotoxicity of chemotherapy in OC.

Recent studies have highlighted the aberrant expression of m6A modification in various cancer and their subtype causing progression, and chemoresistance suggesting its role in development of personalized medicine to overcome drug resistance and high cytotoxicity due to non-specific drugs being targeted by traditional medicine and natural products (23).

**Figure 2:**
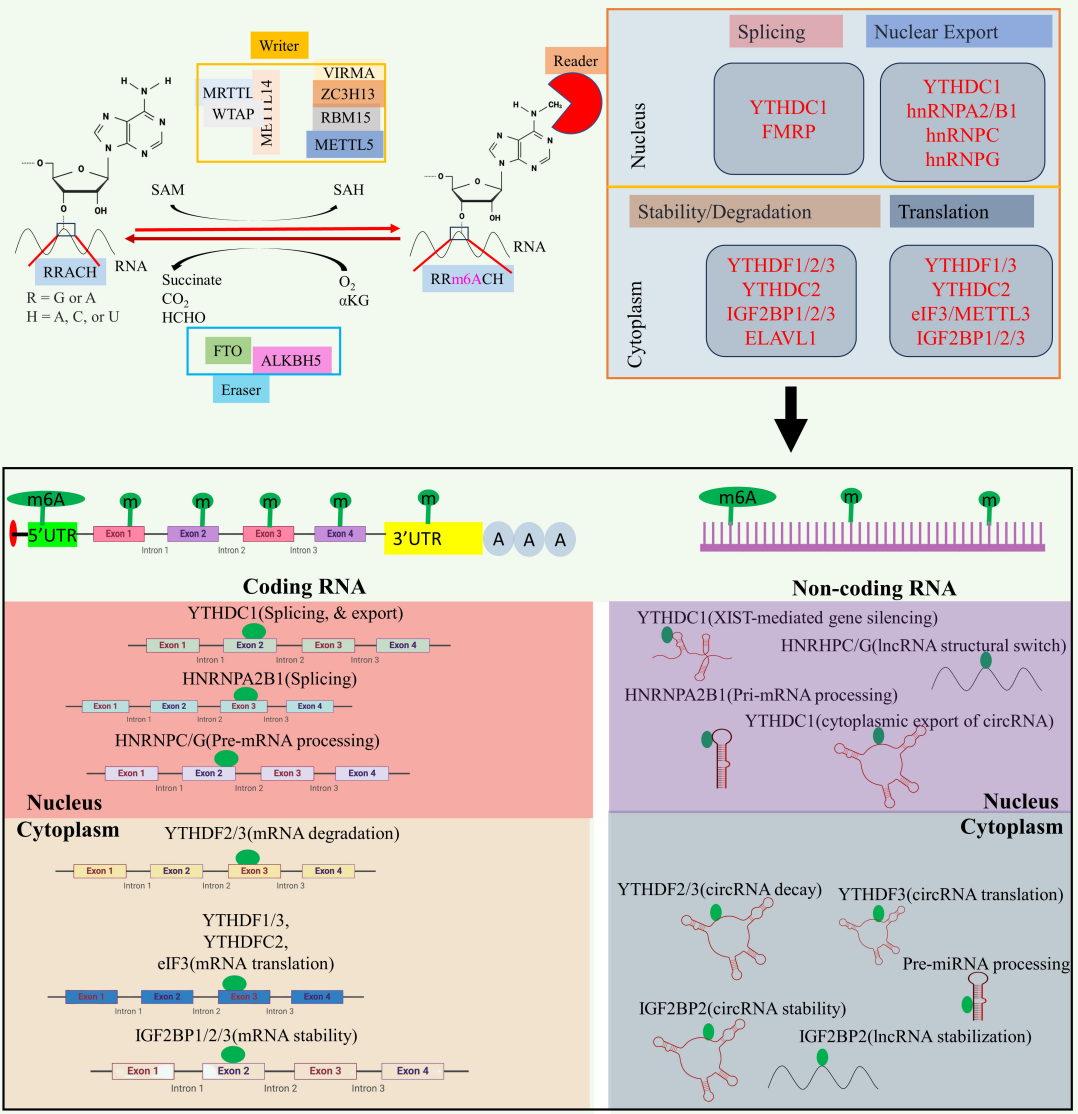
The m6A modification mechanism plays a pivotal role in regulating RNA to modulate various cellular processes. This modification, facilitated by enzymes known as writers, erasers, and readers, involves modifying both mRNA and non-coding RNA. Through this process, m6A modification enhances translation, stability, splicing, and nuclear export of RNA molecules, thereby exerting influence over cellular processes. Created with BioRender.com.

At the molecular level, three primary postsynthetic chemical modifications to DNA, RNA, and protein leads to molecular changes for regulation of different cellular processes. Posttranscriptional mechanism encompassing RNA and non-coding RNAs (ncRNAs) modifications, constitutes a critical mechanism of control at translational level called as epitranscriptomics (24). In the last five decades, the identification of over 100 RNA postsynthetic changes in various types of RNA has expanded our understanding of molecular regulation such as 5-methylcytosine (25, 26), N1-methyladenosine (27–30), and 7-methylguanosine (31–34). Of these, m6A is a most common modification and has significant impact on epitranscriptomic regulation (35–37). N6-methyladenosine, commonly known as m6A, is a reversible prevalent RNA modification characterized via adenosine methylation at nitrogen-6 position at RRACH (R=G or A, and H= A or U or C) sequence. This modification is prevalent across various types of RNA, such as messenger RNA (mRNA), and ncRNA (38, 39). The significance of m6A methylation lies in its crucial role in regulating gene expression and participating in diverse cellular functions. The regulatory dynamics of this modification are orchestrated by a group of proteins classified as “writers,” “erasers,” and “readers” (see **Figure 2**).

Writers are responsible for adding the methyl group, while erasers, includes Fat Mass and Obesity-Associated Protein (FTO) and AlkB Homolog 5 (ALKBH5), play a role in removing the methyl group (40–42). The writers such as Methyltransferase-like 3 (METTL3), and Methyltransferase-like 16 (METTL16) act as a catalyst for m6A modification (43, 44), while methyltransferase-like 14 (METTL14) help to recognize the substrate via METTL3 (45). Wilms’ tumor 1-associating protein (WTAP) promotes the METTL14, and METTL3 heterodimerization, and its movement to nuclear speckle (46) while vir-like m6A methyltransferase associated (VIRMA) guide the methyltransferase to specific RNA (45). RNA-binding motif protein 15 (RBM15) help in recruitment of m6A modification players to specific RNA (47), and zinc finger CCCH-type containing 13 (ZC3H13) help to bridge WTAP to mRNA binding nuclear factor Nito (48). Readers recognizes and binds to the methylated site, thereby influencing subsequent biological phenomenon such as mRNA splicing, and nuclear export [YTH domain-containing proteins C1(YTHDC1) (49–51), and heterogeneous nuclear ribonucleoprotein C (HNRNPC) (52)], promotes translation [YTH N6-methyladenosine RNA binding protein C2 (YTHDC2) (53), and YTH N6-methyladenosine RNA binding protein 1 (YTHDF1) (52)], promotes mRNA stability [insulin-like growth factor 2 mRNA binding protein 1/2/3 (IGF2BP1/2/3) (54)], decreases the mRNA stability or promotes translation [YTH N6-methyladenosine RNA binding protein 3 (YTHDF3) (55)], miRNA processing (heterogeneous nuclear ribonucleoprotein A2/B1 (HNRNPA2B1) (40, 56, 57). Aberrant expression of epitranscriptome has been implicated in different cancers such as lung cancer (58), glioblastoma (59), acute myeloid leukemia (60), colorectal cancer (61), and breast cancer (62). For example, METTL3, VIRMA, FTO, and IGF2BP1 aberrant expression linked to breast cancer, METTL3, FTO, and YTHDF1 disrupted expression promotes lung cancer, and METTL3/14, FTO, and YTHDF2 dysregulated expression promotes acute myeloid leukemia (23). Besides this, m6A modifier such as WTAP, ALKBH5, and YTHDF2 aberrant expression promotes cisplatin resistance, WTAP, and METTL3 dysregulation promotes Adriamycin resistance (63). Several inhibitors of m6A modifier has been identified such as MA2, Dac51, and FB23-2 inhibitors of FTO while IDH2 agonist for METTL3, and MPCH, and U2H1a as inhibitors for METTL3 having low IC50 value which will exhibit low cytotoxicity to overcome OC progression, and chemoresistance (63).

Recent studies have specifically highlighted the abnormal expression of m6A regulators, underscoring m6A methylation role in the incidences OC, and chemoresistance (35).

In the context of this review, we focused on m6A modifications in both coding and noncoding RNAs. We will delve into the molecular processes of these RNA modifications, emphasizing their role as OC prognostic markers and their contributions to OC development, migration, invasion, and development of drug resistance in OC. Additionally, we explored RNA-modified regulators as a promising target for therapeutic strategy for OC, adding a promising dimension to the ongoing efforts in understanding and combating this challenging malignancy.

## 2 m6A RNA modification enzymes as prognostic biomarkers for OC

Biomarkers for determining patient prognosis will help in monitoring patient response to treatment and response to chemotherapy in patients with tumor relapse. Recent studies underscore m6A RNA modification as a prognostic biomarker in OC. An examination of the TCGA database revealed a noteworthy association between elevated WTAP expression and notably inferior overall survival (OS) suggesting writer WTAP as a oncogenic role. The findings of Kaplan−Meier plotter reveal that upregulated alkB homolog 1 (ALKBH1), WTAP, fat mass and obesity associated (FTO), YTHDF1, alkB homolog 1 (ALKBH5), YTHDF3, and YTH N6-methyladenosine RNA binding protein 2 (YTHDF2) as well as decreased expression of METTL14, were linked to poorer OS revealing METTL14 has a tumor suppressor while others were oncogenic m6A modifier (64).

The VIRMA, IGF2BP1, and ZC3H13, prominent N6-methyladenosine modification regulators, independently predict OC prognosis. The robust predictive ability of these parameters highlights their role as significant prognostic biomarkers for OC (65). The m6A modification regulators, such as ZC3H13, insulin-like growth factor 2 mRNA binding protein 2 (IGF2BP2), methyltransferase-like protein 3 (METTL3), VIRMA, and HNRNPC are increased in OC suggesting its role in progression of OC (66). The genes IGF2BP1, VIRMA, HNRNPA2B1, and ELAV-like protein 1 (ELAVL1) are recognized as signature genes for predicting OC prognosis (66).

Decreased expression of seven m6A regulators [METTL14, YTHDC2, FTO, ALKBH5, HNRNPA2B1, VIRMA, and RNA-binding motif protein, X chromosome (RBMX)] was evident in OC tissues sample and in the advanced-stage cohort, suggesting crucial roles in tumor suppressors of OC progression. Patients with upregulated HNRNPA2B1 or downregulated VIRMA had elevated 5-year overall survival rates compared to those of controls. The VIRMA, IGF2BP1, and HNRNPA2B1 are proposed as prognostic biomarker for OC (67). m6A RNA modification regulators, such as VIRMA, HNRNPA2B1, and WTAP, have significant prognostic significance in OC and are linked with the malignant OC development (68). Elevated levels of VIRMA, a writer of m6A, and YTHDC2, a reader of m6A, were linked to an unfavorable prognosis in ovarian patients suggesting they act as a oncogene in OC (69).

The four differentially expressed RNA-modification regulatory genes (DERRG) signature, comprising the Aly/REF export factor (ALYREF), ZC3H13, WTAP, and methyltransferase like 1 (METTL1), were recognized as a self-sufficient prognostic model in OC. This model is valuable for categorizing patients, assessing patient prognosis, and predicting patient response to immunotherapy in patients with OC (70). CACNA1G-AS1, ACAP2-IT1, AC010894.3, and UBA6-AS1 were discovered as prognostic signatures in OC, and each of these genes was associated with methyltransferase-like 5 (METTL5), RBM15, IGF2BP1, and YTH N6-methyladenosine RNA-binding protein C2 (YTHDC1), respectively, suggesting that they regulate the m6A regulatory gene (71). The overexpressed KIAA1429 and YTHDC2 exhibit poor prognosis in OC suggesting its crucial role in OC development (68).

## 3 m6A RNA modifications role in modulating OC progression

**Figure 3:**
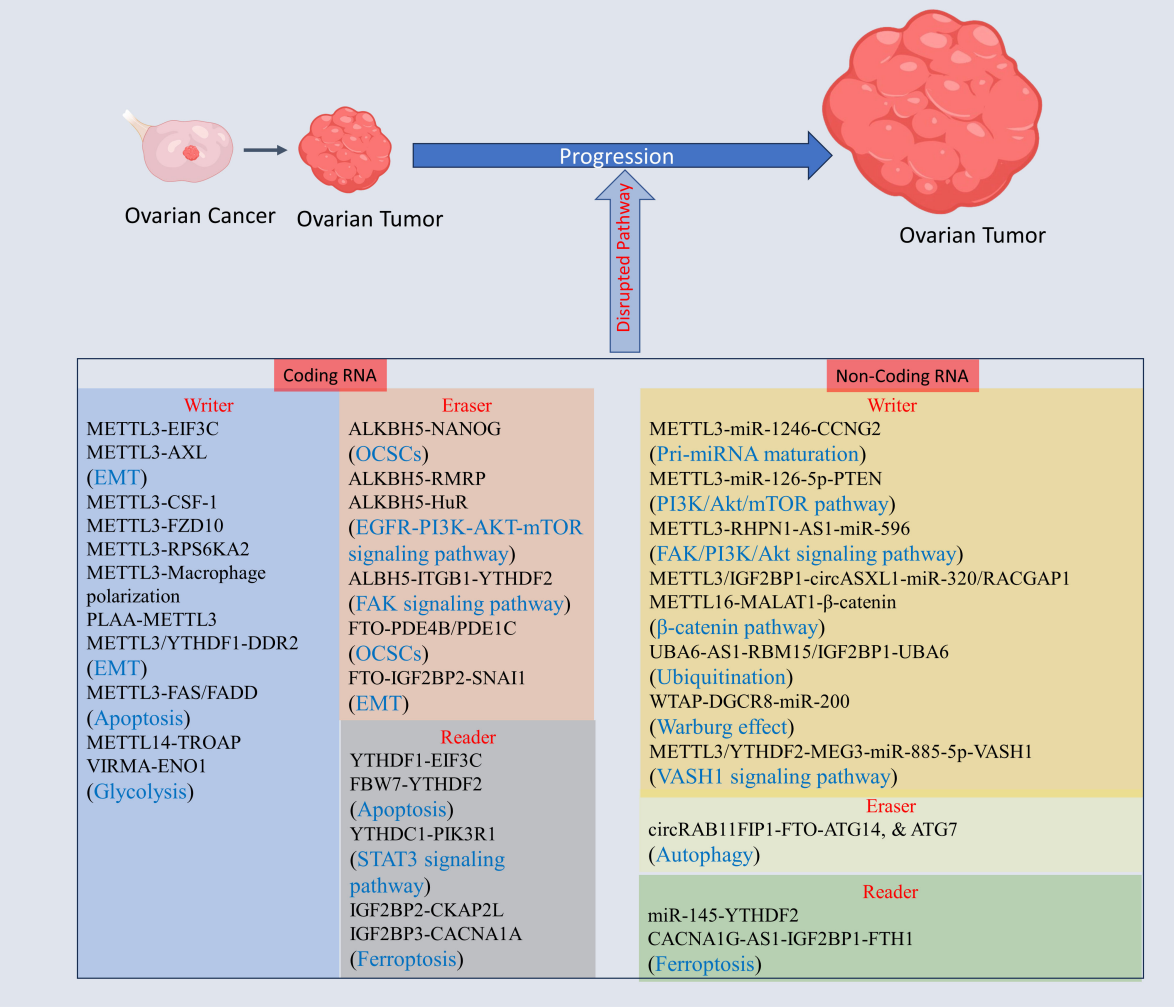
The m6A modification mechanism plays a pivotal role in regulating RNA to modulate various cellular processes. This modification, facilitated by enzymes known as writers, erasers, and readers, involves modifying both mRNA and non-coding RNA. Through this process, m6A modification enhances translation, stability, splicing, and nuclear export of RNA molecules, thereby exerting influence over cellular processes. Created with BioRender.com.

The genes regulating tumorigenesis, invasion, and migration in OC are key players for development of OC, and epitranscriptomic regulation via m6A alteration may have a key role in revolutionizing OC treatments. The m6A modification of mRNA of genes, and non-coding RNA regulating OC progression has gained high importance within few years due to its tremendous potential as a therapeutic target (see **Figure 3**, **Table 1**).

**Table 1:** The role of m6A modification in OC progression, and development.

Category	M6A modification enzyme	mRNA Target Axis	Non-coding RNA Target Axis	References
Writer	METTL3	METTL3-EIF3C	METTL3-miR-1246-CCNG2 (Pri-miRNA maturation)	(29, 51, 72–81)
		METTL3-AXL (EMT)	METTL3-miR-126-5p-PTEN (PI3K/Akt/mTOR pathway),	
		METTL3-CSF-1	METTL3-RHPN1-AS1-miR-596 (FAK/PI3K/Akt signaling pathway),	
		METTL3-FZD10	METTL3/IGF2BP1-circASXL1-miR-320/RACGAP1	
		METTL3-RPS6KA2	METTL3/YTHDF2-MEG3-miR-885-5p-VASH1 (VASH1 signaling pathway)	
		METTL3-Macrophage polarization,		
		PLAA-METTL3		
		METTL3/YTHDF1-DDR2 (EMT)		
		METTL3-FAS/FADD (Apoptosis)		
	METTL14	METTL14-TROAP	–	(82)
	METTL16	–	METTL16-MALAT1-β-catenin (β-catenin pathway)	(83)
	RBM15	–	UBA6-AS1-RBM15/IGF2BP1-UBA6 (Ubiquitination)	(84)
	WTAP	–	WTAP-DGCR8-miR-200 (Warburg effect)	(85)
	VIRMA	VIRMA-ENO1 (Glycolysis)		(86)
Eraser	ALKBH5	ALKBH5-NANOG (OCSCs)	–	(87–90)
		ALKBH5-RMRP		
		ALKBH5-HuR (EGFR-PI3K-AKT-mTOR signaling pathway)		
		ALBH5-ITGB1-YTHDF2 (FAK signaling pathway)		
	FTO	FTO-PDE4B/PDE1C (OCSCs)	circRAB11FIP1-FTO-ATG14, & ATG7 (Autophagy)	(91–93)
		FTO-IGF2BP2-SNAI1 (EMT)		
Reader	YTHDF1	YTHDF1-EIF3C	–	(94)
	YTHDF2	FBW7-YTHDF2 (Apoptosis)	miR-145-YTHDF2	(95, 96)
	YTHDC1	YTHDC1-PIK3R1 (STAT3 signaling pathway)		(97)
	IGF2BP1		CACNA1G-AS1-IGF2BP1-FTH1 (Ferroptosis)	(98)
	IGF2BP2	IGF2BP2-CKAP2L	–	(99)
	IGF2BP3	IGF2BP3-CACNA1A (Ferroptosis)	–	(100)

RPS6KA2, Ribosomal protein S6 kinase alpha-2; EIF3C, Eukaryotic initiation factor 3, subunit; CFZD10, Frizzled class receptor 10; KRT8, Keratin 8; TROAP, Trophinin-associated protein; CKAP2L, cytoskeleton associated protein 2 like; CSF-1, Circulating colony stimulating factor-1; RMRP, RNA component of mitochondrial RNA processing endoribonuclease; CCNG2, Cyclin G2; MALAT1, Metastasis-associated lung adenocarcinoma transcript 1; UBA6-AS1= UBA6 antisense RNA 1; DGCR8, DiGeorge critical region-8; CACNA1G-AS1, CACNA1G antisense RNA 1; FTH1, Ferritin heavy chain 1; PTEN, Phosphatase and TENsin homolog deleted on chromosome 10; UBA6, Ubiquitin-like modifier activating enzyme 6; DDR2, Discoidin domain receptor tyrosine kinase 2; ENO1, Enolase 1; SNAI1, Snail family transcriptional repressor 1; RACGAP1, Rac GTPase activating protein 1.

-, no data available.

### 3.1 m6A RNA modification modulating mRNA to modulate OC progression

METTL3 expression was independently correlated with poorer survival, and increased malignancy in Endometrioid EOC (EEOC). Knocking down METTL3 hindered proliferation and migration, promoting apoptosis compared to that in controls or cells with WTAP or METTL14 knockdown in CRL-11731D, and TOV-112D cell lines. Furthermore, METTL3 knockdown decreased the m6A methylation in genes linked to OC, such as CSF-1, AXL, EIF3C, and FZD10, in CRL-11731D, and TOV-112D cells. This finding suggested that METTL3-driven m6A modification is distinct from that of WTAP and METTL14 (72). Another study suggests METTL3 knockdown decreased Cyclin D1 along with reduced AKT phosphorylation (101). RPS6KA2 and JUNB were strongly linked with unfavorable prognosis of OC, and there was a positive correlation observed between RPS6KA2 and METTL3 in OC suggesting that RPS6KA is regulated through METTL3-dependent m6A modification (73). Silencing METTL3 in the endometrioid OC cell line COV362 significantly reduced proliferation, and induces G0/G1 cell cycle arrest to enhance cell death (102). OC cell growth increased in METTL3-cKO mice. OC progression was characterized by a change from macrophage polarization from M1 to M2, indicating downregulation in M1 and upregulation in M2 polarization (74). The METTL3, METTL14, IGF2BP2, FTO, and ELF3 have dysregulated expression in EOC, with METTL3 exhibiting highest upregulation. METTL3 silencing induces G0/G1 phase arrest and apoptosis. Conversely, METTL3 overexpression showed reverse effect. Sulforaphene (Sul) reversed METTL3 overexpression, reducing EOC cell viability and promoting apoptosis. Mechanistic study shows that knockdown of METTL3 result in FAS/FADD pathway activation, and altering Bax/Bcl-2 pathway. Sul promotes the apoptosis via decreasing the METTL3 expression, and inducing subsequent apoptosis pathway along with increasing the expression of IGF2BP2 and fas cell surface death receptor (FAS) and downregulating KRT8 (80). PLAA showed reduced expression in highly metastatic OC. Mechanistic study shows PLAA promotes METTL3 degradation, leading to destabilization of TRPC3 which regulate intracellular calcium flux to inhibit metastasis in OC (103). The silencing of METTL3/YTHDF1 inhibit OC progression and mechanistic study reveals that METTL3/YTHDF1 axis enhanced the expression of the tumor-promoting DDR2 to foster the progression of OC (75). METTL3 is also reported to induce epithelial to mesenchymal transition (EMT) by enhancing AXL expression (76). METTL3 is shown to regulate various biological process to promote the OC progression, therefore, METTL3 inhibition will help to overcome OC mortality. The overexpressed KIAA1429(VIRMA) promotes the OC proliferation and inhibit necrosis. Mechanistic study shows KIAA1429 stabilizes the ENO1 mRNA in m6A dependent way to promote glycolysis, and proliferation of OC (86). In EOC tissues, both METTL14 expression and m6A RNA methylation levels were notably lower than those in normal tissues (82). A mechanistic study revealed that METTL14 functions as an inhibitor of EOC proliferation through the suppression of TROAP expression through a mechanism dependent on m6A RNA methylation (82). On contrary, the METTL14 was overexpressed in EOC tissues, and induces proliferation, migration, and invasion in A2780, and SKOV3 EOC cell line (104).

ALKBH5 is overexpressed in OC tissue whereas it is downregulated in cell lines. Similarly, the tumor microenvironment Toll-like receptor 4 (TLR4) exhibited this trend. Coculturing OC cells with M2 macrophages led to high expression of both ALKBH5 and TLR4. A mechanistic study revealed that upregulation of TLR4 activated nuclear factor kappa B (NF-κB) axis, causing upregulated ALKBH5, increased m6A levels and increased NANOG expression, promoting the aggressiveness of OC (87). ALKBH5 was overexpressed in EOC. In SKOV3 cells, the suppression of ALKBH5 heightened autophagy and restrained the proliferation and invasion (89). Mechanistic experimental studies reveal that ALKBH5 interacted with HuR, activating EGFR-PIK3CA-AKT-mTOR axis and promoting stabilization of BCL-2, promoting Bcl-2 and Beclin1 interaction (89). These findings further support the finding that ALKBH5 is dysregulated in OC (105). Elevated ALKBH5 expression in OC is induced by a hypoxic microenvironment, and upon inhibiting hypoxia-inducible factor (HIF)-1, ALKBH5 expression decreases concomitantly with a reduction in HIF-1 mRNA expression. ALKBH5 is overexpressed in human OC to promote OC growth and migration. A mechanistic study showed that ALKBH5 upregulates RMRP expression through demethylation and that knocking down RMRP in the OVCAR3 and SKOV-3 cell lines reduces cell growth and migration (88). ALKBH5 overexpression induced by HIF-1α promotes EOC metastasis via targeting Integrin beta 1 (ITGB1) to block YTHDF2 dependent ITGB1 degradation to induce FAK phosphorylation at Tyr397 to trigger Src kinase phosphorylation at Tyr416 (90). In OC and OC stem cells (OCSCs), FTO expression is decreased. FTO overexpression hinders the self-renewal characteristics of OCSCs and suppresses *in vivo* tumorigenesis through the demethylase activity of FTO. Mechanistically, FTO inhibits phosphodiesterases 4B (PDE4B) and 1C (PDE1C) via demethylase activity via m6A modification, resulting in cAMP accumulation and reduced stemness in OC and tumor initiation (91). FTO is reported to induce oxidative stress and apoptosis in OC, leading to suppressed tumor in nude mice (106). FTO decreased expression promotes OC progression. Mechanistic study shows FTO inhibit SNAI1 stability in an IGF2BP2 dependent manner to inhibit EMT suggesting FTO-IGF2BP2-SNAI1 axis role in OC progression (92). Therefore, FTO agonist can be used to overcome OC progression.

YTHDF1 is upregulated in OC, and linked to unfavorable prognostic outcomes in OC patients. A mechanistic study showed that YTHDF1 enhances EIF3C translation via interacting with m6A-methylated EIF3C mRNA, suggesting that YTHDF1-EIF3C pathway is relevant for OC progression (94). FBW7 is downregulated and promote OC progression. Mechanistic study shows that FBW7 protect pro apoptotic genes BMF degradation via inhibiting YTHDF2 (95). In OC, m6A modification and IGF2BP2 expression are significantly elevated. IGF2BP2 overexpression enhanced OC growth, migration, and invasion. A mechanistic study shows that IGF2BP2 promoted translation of CKAP2L in m6A methylation dependent way without altering mRNA or protein stability. Further study revealed that overexpressing CKAP2L promoted the progression of OC cells with IGF2BP2 knockdown (99). YTHDC1 downregulated in OC while its overexpression inhibited OC development. Mechanistic study reveal YTHDC1 promotes PIK3R1 stability which decreases GANAB via STAT3 pathway (97). The overexpressed IGF2BP3 promotes the OC proliferation via inhibiting ferroptosis. Mechanistic study shows IGF2BP3 target CACNA1A. The silencing of CACNA1A promotes ferroptosis due to aberrant intracellular Ca^2+^ leading to high ROS suggesting IGF2BP3-CACNA1A axis in OC progression (100).

### 3.2 m6A RNA modification modulating non-coding RNA to modulate OC progression

In eukaryotic cells, ncRNAs typically lack ability to encode proteins. Instead, they carry out biological phenomenon at the RNA level. Traditionally, ncRNAs recognized as posttranscriptional regulators of gene expression. However, recent insights into RNA modifications have broadened their regulatory influence on gene expression. A notable example is m6A, a reversible epitranscriptomic alteration occurring at N6 of adenosine. This alteration is crucial in regulating RNA degradation, RNA splicing, and other biological processes. The scientific literature has previously detailed the molecular pathways that govern the role of m6A modification regulating expression of ncRNA. It is noteworthy that the levels of m6A alteration and m6A regulators expression are intricately controlled by ncRNAs (38). The ncRNA are broadly categories into housekeeping ncRNA, and regulatory ncRNA. Ribosomal RNA (rRNA), transfer RNA (tRNA), small nuclear RNA (snRNA) are known as housekeeping ncRNA while microRNA(miRNA), circular RNA (circRNA), and long non-coding RNA (lncRNA). The miRNA, circRNA, and lncRNA are classified based on their length such as miRNA are 21-23 nucleotides, circRNA are 100-10000 nucleotides, and lncRNA are greater than 200 nucleotides (107).

METTL3 was overexpressed, and hypomethylated in OC tissues and cells and displays a negative correlation with overall survival. Downregulated METTL3 hindered growth and migration of OC to induce cell death. Conversely, overexpression of METTL3 shows opposing phenotype. The underlying mechanism involved METTL3 promoting OC via targeting miR-1246, leading to suppression of CCNG2. Additionally, elevated METTL3 levels downregulated CCNG2, fostering tumors growth in mice (78). miR-126-5p is overexpressed in OC to promote proliferation, migration, and invasion. A mechanistic study revealed that METTL3 promotes miR-126-5p maturation through pri-miR-126-5p’s m6A methylation, which directly binds to PTEN, leading to PI3K/Akt/mTOR pathway activation (79). RHPN1-AS1 augments growth and migration of EOC by functioning as a competing endogenous RNA (ceRNA), where it sequesters miR-596. This interaction results in increased LETM1, leading to FAK/PI3K/Akt pathway activation. Further research reveal that silencing METTL3 decreased RHPN1-AS1 expression, resulting in reduced stability of RHPN1-AS1, suggesting that RHPN1-AS1 regulation is METTL3-dependent on m6A modification (77). The circASXL1 was identified to promote the OC progression via miR-320/RACGAP1 axis. Mechanistic study reveals that METTL3/IGF2BP1 promotes the circASXL1stability in m6A dependent manner (108). METTL16 is downregulated in EOC tissue, and MALAT1 is upregulated in EOC tissue. Further study revealed that METTL16 suppressed EOC growth by facilitating MALAT1 degradation. In turn, upregulated β-catenin to facilitate its nuclear transport in EOC cells, suggesting that the METTL16-MALAT1-β-catenin axis inhibits EOC progression through METTL16 (83). Overexpressed WTAP promoted OC proliferation and invasion. Further study revealed that WTAP interaction with DGCR8 to modulate the microRNA-200 (miR-200) expression in a m6A-modification dependent manner, and the glycolysis enzyme hexokinase 2 (HK2) was found to be positively regulated by miR-200. WTAP was found to be positively regulated by HIF-1α under hypoxia in OC (85).

In EOC tissues, YTHDF2 expression was notably upregulated compared to that in normal ovarian tissues. Functional investigations shows that YTHDF2 role in enhancing EOC growth and migration while reducing overall 6-methyladenine (m6A) mRNA levels. A mechanistic study showed that miR-145 levels were inversely correlated with YTHDF2 levels, and YTHDF2 was discovered as a target of miR-145 (96). The long noncoding RNA (lncRNA) CACNA1G-AS1 was shown that it increases the growth, and migration of OC. Further support for these findings was obtained by knocking down CACNA1G-AS1, which reduced OC tumorigenesis *in vivo*. A mechanistic study showed that CACNA1G-AS1 upregulates FTH1 expression via the IGF2BP1 axis, inhibiting ferroptosis through ferritinophagy regulation (98). UBA6-AS1 was shown that it inhibits the growth, invasion, and migration of OC through its interaction with UBA6. A mechanistic study showed that UBA6-AS1 increased UBA6 mRNA’s m6A methylation by enlisting RNA binding motif protein 15 (RBM15) for methylation. Additionally, insulin-like growth factor 2 mRNA binding protein 1 (IGF2BP1) was found as reader protein for UBA6-AS1-RBM15 dependent UBA6 mRNA’s m6A modification, thereby enhancing its stability (84). The lncRNA MEG3 was found to be downregulated in OC to promote OC malignant phenotype. Mechanistic study shows increasing the levels of MEG3 inhibited the breakdown of VASH1 via functioning as a suppressor of miR-885-5p. Further study shows that YTHDF2 enhances MEG3 degradation via METTL3 dependent (81). circRAB11FIP1 was overexpressed in SKOV3 OC cell lines to promote OC progression, Mechanistic study shows that DSC1 interact with circRAB11FIP1 to regulate its expression, and sponge miR-129 to regulate ATG14 and ATG7 to promote autophagic flux. Further study shows circRAB11FIP1 regulate ATG14 and ATG7 via FTO dependent m6A mRNA modification (93).

## 4 The role of m6A RNA modifications in modulating drug resistance in OC

**Figure 4:**
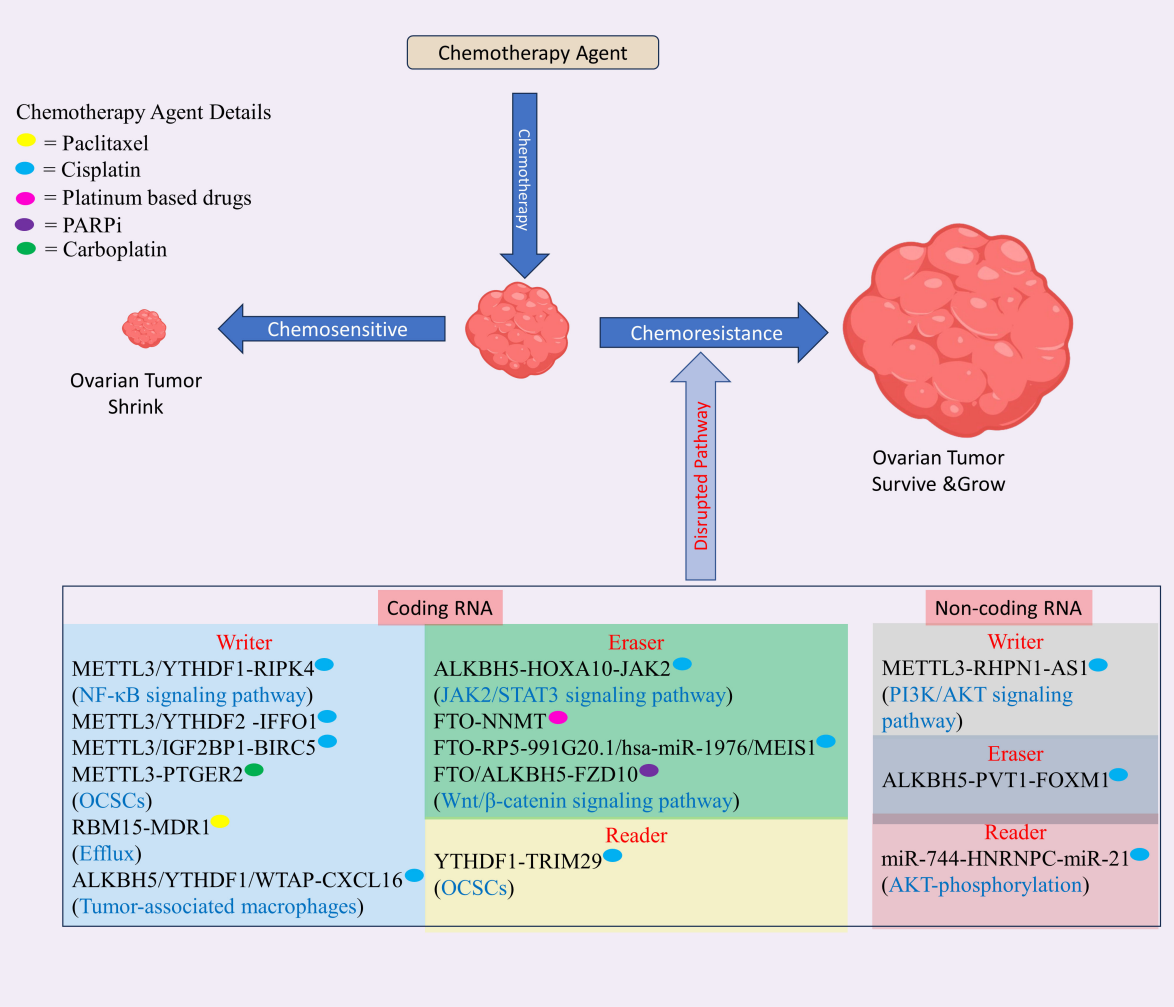
The role of m6A modification in drug resistance in ovarian cancer (OC) involves modifications occurring in both mRNA and non-coding RNA. These modifications regulate the resistance of various drugs used in OC treatment, including platinum-based compounds like cisplatin and carboplatin, and PARP inhibitors. This regulatory mechanism disrupts normal pathways, contributing to drug resistance in OC. Created with BioRender.com.

Despite an initially promising response to initial treatment, the chemotherapy resistance diminishes effectiveness of chemotherapy, resulting in increased relapse rates and reduced long-term survival in patients with OC. Research indicates that up to two out of three of higher stage OC patients experience relapse of tumor within eighteen months, regardless of initial therapy (109). Recent discovery shows that m6A modification has a significant contribution in drug resistance development at mRNA, and non-coding RNA level in OC (see **Figure 4**, **Table 2**).

**Table 2:** The Role of m6A modification in drug resistance in OC.

Category	M6A modification enzyme	Chemotherapy drug	mRNA Target Axis (Pathway)	Non-coding RNA Target Axis (Pathway)	References
Writer	METTL3	Cisplatin	METTL3/YTHDF1-RIPK4 (NF-κB signaling pathway)	METTL3-RHPN1-AS1 (PI3K/AKT signaling pathway)	(110–112)
			METTL3/YTHDF2 -IFFO1		
			METTL3/IGF2BP1-BIRC5		
		Carboplatin	METTL3-PTGER2 (OCSCs)	–	(113)
	RBM15	Paclitaxel	RBM15-MDR1(Efflux)	–	(114)
	WTAP	Cisplatin	ALKBH5/YTHDF1/WTAP-CXCL16 (Tumor-associated macrophages)	–	(115)
Eraser	ALKBH5	Cisplatin	ALKBH5-HOXA10-JAK2 (JAK2/STAT3 signaling pathway)	ALKBH5-PVT1-FOXM1	(116, 117)
	FTO	Platinum	FTO-NNMT	–	(118)
		Cisplatin	FTO-RP5-991G20.1/hsa-miR-1976/MEIS1	–	(119)
		PARPi	FTO/ALKBH5-FZD10(Wnt/β-catenin signaling pathway)	–	(120)
Reader	YTHDF1	Cisplatin	YTHDF1-TRIM29 (OCSCs)	–	(121)
	HNRNPC	Cisplatin	–	miR-744-HNRNPC-miR-21 (AKT-phosphorylation)	(122)

RIPK4, Receptor interacting protein kinase 4; RHPN1-AS1, Rhophilin rho GTPase binding protein 1 antisense RNA 1; IFIO1, Intermediate filament family orphan 1; PTGER2, Prostaglandin E receptor 2; CXCL16, CXC chemokine ligand 16; NNMT, Nicotinamide N-methyltransferase; MEIS1, Meis Homeobox 1; PVT1, Plasmacytoma variant translocation 1; FOXM1, Forkhead box protein M1.

-, no data available.

### 4.1 m6A RNA modification modulating mRNA to modulate drug resistance in OC

YTHDF1, YTHDF2, WTAP, FTO, and ALKBH5 exhibited elevated expression levels in OC and were confirmed to be significant prognostic risk factors associated with decreased overall survival (OS) suggesting its oncogenic role in OC development. Downregulated YTHDC1 and upregulated RBM15 were linked to the metastatic potential of OC. In the group resistant to chemotherapy, HNRNPC, METTL3, and RBM15 exhibited decreased expression, and RMBX and METTL14 showed increased expression suggesting their role as a inhibitors, and promoter of resistance in OC respectively. Elevated HNRNPC expression reliably indicated a favorable response to paclitaxel in patients with OC (123).

RHPN1-AS1 and METTL3 are upregulated in OC, and a mechanistic study showed that METTL3 enhances stability of RHPN1-AS1 through m6A methylation, leading to overexpressed phosphorylated Akt and PI3K to confer cisplatin resistance in OC. Notably, overexpressed RHPN1-AS1 enhances cell growth, migration, invasion, and *in vivo* tumor proliferation (111). RIPK4 was upregulated in OC, fostering cisplatin resistance and tumor progression. Through mechanistic investigations, it was discovered that YTHDF1 enhances RIPK4 expression in METTL3-dependent fashion. This enhancement occurs due to inhibition of degradation of YTHDF1 mRNA. Subsequently, overexpressed RIPK4 leads to the NF-κB phosphorylation, ultimately triggering tumorigenesis and fostering resistance to cisplatin in EOC (110). PTGER2 is overexpressed in OC, and silencing PTGER2 diminishes the stemness of OC cells; reduces CD44 and CD133 expression; inhibits carboplatin resistance, migration, and invasion; increases DNA damage, as indicated by elevated γH2AX levels; and impairs EMT-related proteins such as vimentin, myc, and cyclin D1 (CCND1). Further study revealed that PTGER2 expression was elevated through METTL3-mediated m6A modification (113). IFFO1 is downregulated in OC to confer cisplatin resistance, tumor progression, and metastasis, and overexpressed IFFO1 hinders the β-catenin translocation to nucleus, resulting in reduced metastasis and enhanced sensitivity to cisplatin. A mechanistic study showed that histone deacetylase 5 (HDAC5) recruitment suppressed IFFO1 expression through yin yang 1 (YY1) and that the METTL3/YTHDF2 axis controlled IFFO1 stability through m6A modification (112).

RBM15 was discovered to be overexpressed in OC and linked with unfavorable prognosis. Silencing RBM15 was shown to decrease paclitaxel resistance and vice versa. A mechanistic study showed that RBM15 silencing reduced the m6A methylation of multidrug resistance 1 (MDR1) mRNA and that TGF−β pathway activation results in inhibition of RBM15, suggesting that TGF−β/RBM15/MDR1 is a regulatory mechanism that confers paclitaxel resistance in OC (114). Tumor-associated macrophages (TAMs) coculturing with OC cells enhance cisplatin resistance via increasing CXCL16 expression. Silencing of CXCR6 in OC or CXCL16 in TAMs inhibited cisplatin resistance observed in cells cocultured with TAMs, suggesting that CXCL16 contribute to cisplatin resistance development. A further study showed that silencing CXCL16 downregulated YTHDF1/WTAP and upregulated ALKBH5, and enhancing the expression of WTAP increased cisplatin resistance in OC, suggesting that cisplatin resistance is mediated through YTHDF1, WTAP, and ALKBH5 and can be targeted to overcome chemoresistance in OC (115).

ALKBH5 is found to be upregulated in cisplatin resistant OC to induce cisplatin resistance in OC via ALKBH5-HOXA10 loop which target JAK2 to activate JAK2/STAT3 pathway (117). FTO expression was downregulated in platinum-resistant OC, while NNMT expression was enhanced upon FTO overexpression. The sensitivity of FTO-overexpressing cells to platinum was restored upon NNMT inhibitor treatment or by silencing NNMT, suggesting that FTO mediated platinum resistance in OC (118). The MEIS1, and RP5-991G20.1 expression were significantly lower in the knockout of FTO group, while the hsa-miR-1976 level was significantly greater and negatively correlated with the FTO level. These findings suggested that FTO alterations influence RP5-991G20.1/hsa-miR-1976/MEIS1 signaling pathway. Patients with cisplatin resistance (PFS < 6 months) displayed elevated hsa-miR-1976 expression, in contrast with the decreased expression in cisplatin-sensitive patients (PFS > 6 months). Furthermore, RP5-991G20.1, FTO, and MEIS1 elevated in tumors received from patients with clinically described cisplatin sensitivity (PFS > 6 months) and reduced in tumors received from patients with clinically described cisplatin resistance (PFS < 6 months). These findings suggested that the FTO/RP5-991G20.1/hsa-miR-1976/MEIS1 axis regulate cisplatin resistance in OC. More research revealed that FTO knockdown markedly increased growth and resistance of A2780 OC cell line to cisplatin and PPARis. These studies reveal that FTO constrains the proliferation and drug resistance of OC cells, underscoring its pivotal role in reversing OC resistance (119). m6A modification was found to promote resistance to PARP inhibitors (PARPis) in BRCA-mutant EOC by enhancing Wnt/β-catenin axis through FZD10. A mechanistic study showed that silencing FTO and ALKBH5 elevated the FZD10 mRNA’s m6A methylation and decreased sensitivity to PARPis (120). Therefore, FTO, and ALKBH5 agonist are crucial to overcome PARPi resistance in OC.

The overexpressed BIRC5 promotes cisplatin resistant in OC. Mechanistic study shows METTL3/IGF2BP1 promotes BIRC5 mRNA to confer cisplatin resistance in OC (124). The TRIM29 was overexpressed in cisplatin-resistant OC and enhanced the CSC like phenotype in cisplatin resistant OC. Mechanistic study shows YTHDF1 promotes the TRIM29 translation in a m6A dependent manner to confer cisplatin resistance in OC (121).

### 4.2 m6A RNA modification influences non-coding RNA to modulate drug resistance in OC

The m6A modification of ncRNA also affect the drug resistance in ovarian cancer leading to failure of current chemotherapeutic treatment. Therefore, we had scrutinize the m6A modification and ncRNA related discoveries in current section. LINC02489 expression was decreased in tissue samples from metastatic and chemoresistant OC. Mechanistic study reveals that LINC02489 hinders the invasion and migration of chemoresistant OC by boosting its m6A modification and increasing PKNOX2 expression. Additionally, it regulates OC cell invasion through the PTEN/mTOR pathway, affecting the paclitaxel resistance in SKOV3. These finding suggest m6A modification regulate non-coding RNA (125).

ALKBH5 exhibited increased expression in OC. The silencing ALKBH5 resulted in reduced tumor growth and invasion, while enhancing sensitivity to cisplatin, docetaxel, and 5-FU. Mechanistically, ALKBH5 was found to promote the stability of PVT1 RNA, which, in turn, regulated FOXM1, influencing both chemosensitivity and malignant characteristics in OC (116). miRNA-744 was discovered to be downregulated in OC, while its overexpression induced apoptosis in SKOV3, OVCAR3 and cisplatin-sensitive and cisplatin-resistant A2780 cell lines. A mechanistic study showed that miR-744 downregulates HNRNPC, and nuclear factor one X (NFIX) and HNRNPC knockdown downregulate miR-21, which suppressed programmed cell death 4 (PDCD4), and PTEN suggesting its role in development of combinatorial therapy (122).

## 5 Clinical significance of m6A RNA modification in OC

OC remains one of the most challenging gynecological malignancies to diagnose and treat effectively. Despite advancements in treatment modalities, including surgery and chemotherapy, the prognosis for OC patients often remains poor. There’s a critical need for new treatment targets and markers to enhance clinical care. In recent years, epitranscriptomic, particularly m6A RNA modification, has emerged as a key regulator of gene expression and has garnered significant attention in OC research. In this section, we discuss clinical significance of m6A modification in OC and its implications for diagnosis, prognosis, and treatment.

Recent studies have demonstrated aberrant m6A RNA modification patterns in OC tissues versus normal ovaries. These dysregulated m6A modifications have been associated with alterations in gene expression profiles that contribute to ovarian tumorigenesis. Importantly, m6A RNA modifications have shown promise as potential diagnostic biomarkers for OC (72, 95). Detection of specific m6A modified RNA transcripts in blood or tissue samples may facilitate the early detection of OC, thereby improving patient outcomes through timely intervention (79, 126, 127).

Disruption of m6A RNA modification correlates with OC advancement and metastasis. Elevated levels of m6A modification enzymes, including METTL3 and METTL14, have been linked to unfavorable clinicopathological features and poor prognoses among OC patients. Conversely, reduced expression of the m6A demethylase FTO has been correlated with improved survival outcomes. YTHDF1 has been identified as a promoter of OC cell tumorigenesis by regulating eIF3C translation via m6A-dependent way, thereby influencing global protein translation in OC (94). Furthermore, Han et al. demonstrated a significant increase in WTAP expression in ovarian tissues, with its high expression correlating with cell cycle regulation and MYC targeting (64). Aberrant RNA modification may contribute to tumor development. Jie Li et al. revealed that miR-145-mediated inhibition of YTHDF2 regulates the proliferation, apoptosis, and migration of OC (96). Takeshi Fukumoto et al. discovered that m6A modification of FZD10 regulate PARP inhibitor resistance (120). Zhang et al. found an 18% mutation rate and high expression of ZC3H13 in OC samples, with its expression negatively correlated with prognosis. METTL16 was found to be under expressed in OC tissues and positively correlated with prognosis, especially in patients under 60 years old, those in stage III-IV, and those with tumors (68). Dysregulation of m6A modification regulators has been observed in OC tissues compared to normal ovaries. Patients exhibiting high expression of KIAA1429 and YTHDC2 were found to have poorer prognoses (69). METTL3 enhanced miR-126-5p maturation, accelerating OC progression (79). Additionally, another study demonstrated that METTL3 facilitated OC growth and invasion by activating EMT (76). ALKBH5 expression was higher in EOC tissues than in normal ovarian tissues, suggesting its oncogenic potential in EOC (89). The “reader” protein IGF2BP1 enhances SRC/MAPK-driven invasive growth of OC cells, and overexpressed IGF2BP1 is linked with unfavourable prognosis in OC patients (128, 129). These findings suggest that evaluating m6A RNA modification status could serve as a prognostic indicator in OC, aiding in risk stratification and treatment decision-making.

Targeting dysregulated m6A RNA modification pathways hold promise as a novel therapeutic strategy for OC. Small molecule targeting m6A modification complex components have demonstrated efficacy in preclinical studies, inhibiting OC cell proliferation, migration, and invasion (130, 131). Moreover, modulating m6A RNA levels enhances OC cell sensitivity to therapies. YTHDF1 recruitment to m6A-modified TRIM29 accelerates translation in cisplatin-resistant OC cells, underscoring its therapeutic potential (121). These findings highlight the potential of m6A RNA modification-targeted therapies as adjuvant treatments for OC, offering new avenues for personalized medicine approaches.

Overall, the clinical significance of m6A RNA modification in OC is becoming increasingly evident. Dysregulated m6A RNA modification patterns have diagnostic, prognostic, and therapeutic implications in OC, presenting chances for novel biomarker and targeted therapy development. Further studies is warranted to elucidate underlying mechanisms of m6A modification dysregulation in OC and to translate these findings into clinical practice for improved patient outcomes.

## 6 Conclusion and future direction

Comprehensive exploration of m6A RNA modifications in OC reveals a nuanced landscape in which molecular intricacies intersect with clinical challenges. The multifaceted role of m6A modifications on both coding and ncRNAs has emerged as a critical determinant of the initiation, progression, and potential therapeutic intervention for OC. This review underscores the dynamic and reversible nature of m6A modifications, shedding light on their regulatory roles in gene expression at various stages of mRNA and noncoding RNA (ncRNA) life. The orchestrated interplay between writers, erasers, and readers of m6A modifications offers a compelling narrative of how these molecular actors contribute to OC development, migration, invasion, and emergence of drug resistance. The m6A-mediated modification genes YTHDF2, IGF2BP2, RBMX, METTL1, ALKBH5, METTL3, YTHDC1, METTL5, HNRNPC, METTL14, WTAP, YTHDF1, FTO, ALKBH1, YTHDF3, YTHDC2, IGF2BP1, VIRMA, ZC3H13, KIAA1429, HNRNPA2B1, ELAVL1, ALYREF, RBM15, and FTO are prognostic markers in OC. Similarly, METTL3, METTL14, ALKBH5, FTO, ELF3, IGF2BP2, IGF2BP3, YTHDF2, YTHDFC1, and YTHDF1 have been shown to modulate OC progression at mRNA level while METTL3, METTL16, WTAP, RBM15, FTO, IGF2BP1, and YTHDF2 interact with non-coding RNA to regulate OC progression. The m6A alteration role in drug resistance is very poorly understood compared to that in progression, and METTL3, RBM15, WTAP, FTO, ALKBH5, YTHDF1, METTL14, HNRNPC, RBMX, IGF2BP1, YTHDC1, and YTHDF2 have been shown to promote chemoresistance in OC at mRNA level via disruption of signaling axis such as FTO disrupt RP5-991G20.1/hsa-miR-1976/MEIS1 signaling pathway to modulate chemoresistance in OC and ALKBH5, and HNRNPC interact with non-coding RNA to confer chemoresistance in OC.

Despite significant progress in m6A modification role in OC, several challenges persist. The heterogeneity of OC poses a significant obstacle, as distinct tumor subtypes exhibit diverse m6A modification profiles. Additionally, the lack of standardized methodologies for detecting and quantifying m6A modifications hampers comparative analyses and the establishment of uniform diagnostic criteria. Similarly, some studies are not conclusive due to contradictory finding such as FTO, and ALKBH5 (64), and VIRMA, and YTHDC2 (69) are upregulated but other studies show its downregulation in OC (67). Similarly, METTL14 found as both inhibitors and promoters of OC progression (82, 104). RBM15 expression was also contradictory due to studies on different sample such as TCGA data analysis shown RBM15 downregulation while cell-based studies shows RBM15 increased expression (114, 123). Therefore, more robust studies are required to establish their role in OC development.

The m6A modification signals promising avenues for addressing different cancer types. Exploring m6A modification regulators or inhibitors offers promising therapeutic paths for OC. While certain inhibitors targeting m6A methylation have demonstrated positive effects on cancer progression (132, 133), there is a need for further investigation into novel therapeutic strategies involving m6A RNA methylation for the treatment of OC.

This review highlights the potential therapeutic avenues that could arise from targeting m6A modifications in OC. Given m6A research importance for gene expression regulation, developing strategies to modulate these modifications may open new frontiers in OC treatment. Furthermore, understanding the crosstalk between m6A modifications and other molecular pathways implicated in OC could unveil synergistic therapeutic approaches.

Integrating m6A modification data with other omics data holds great promise, and comprehensive multiomics analyses could provide a holistic view of the molecular landscape in OC, offering a more nuanced understanding of the disease and identifying novel therapeutic vulnerabilities.

In conclusion, while m6A modification’s role in OC has been revealed to a significant extent, the related literature is far from complete. Addressing current challenges and seizing future opportunities will propel researchers toward a more comprehensive understanding of the role of m6A in OC, fostering the innovative and effective therapeutic strategies development for this complex and challenging malignancy.

## Author contributions

PG: Conceptualization, Data curation, Formal analysis, Funding acquisition, Investigation, Methodology, Project administration, Resources, Software, Supervision, Validation, Visualization, Writing – original draft, Writing – review & editing. SA: Writing – original draft, Writing – review & editing.
